# The Effect of Exercise Prescription on the Human Gut Microbiota and Comparison between Clinical and Apparently Healthy Populations: A Systematic Review

**DOI:** 10.3390/nu15061534

**Published:** 2023-03-22

**Authors:** Alexander N. Boytar, Tina L. Skinner, Ruby E. Wallen, David G. Jenkins, Marloes Dekker Nitert

**Affiliations:** 1 School of Human Movement and Nutrition Sciences, The University of Queensland, Brisbane, QLD 4072, Australia; 2 School of Health, University of the Sunshine Coast, Maroochydore, QLD 4558, Australia; 3 Applied Sports Science Technology and Medicine Research Centre, Swansea University, Wales SA1 8EN, UK; 4 School of Chemistry and Molecular Biosciences, The University of Queensland, Brisbane, QLD 4072, Australia

**Keywords:** physical activity, gut microbiota, microbiome, exercise intensity, exercise frequency, exercise duration

## Abstract

This study systematically reviewed all human longitudinal exercise interventions that reported changes in the gut microbiota; frequency, intensity, duration and type of exercise were assessed to determine the influence of these variables on changes to the gut microbiota in both healthy individuals and clinical populations (PROPERO registration: CRD42022309854). Using PRISMA guidelines, trials analysing gut microbiota change with exercise interventions were included independent of trial randomisation, population, trial duration or analysis technique. Studies were excluded when microbiota abundance was not reported or when exercise was combined with other interventions. Twenty-eight trials were included, of which twelve involved healthy populations only and sixteen involved mixed or clinical-only populations. The findings show that participation in exercise of moderate to high-intensity for 30–90 min ≥3 times per week (or between 150–270 min per week) for ≥8 weeks is likely to produce changes in the gut microbiota. Exercise appears to be effective in modifying the gut microbiota in both clinical and healthy populations. A more robust methodology is needed in future studies to improve the certainty of the evidence.

## 1. Introduction

The human gut microbiota has been implicated in numerous aspects of health and wellbeing, including disease risk and healthy aging [1,2,3,4]. There is a growing body of literature describing how diet, prebiotic and probiotic supplementation and more recently, exercise can potentially improve health outcomes by modifying and protecting the gut microbiota [5,6,7]. Changes to the gut microbiota can be assessed using three broad classifications: (1) diversity, which can be determined through a variety of metrics and generally considers a combination and weighting of—but not limited to—the species number, richness and spread of different microbes available within a sample (alpha diversity), or the similarity or dissimilarity between two samples (beta diversity), (2) relative abundance, which considers the proportion of a sample that a particular microbe contributes to and can be measured at multiple levels of taxonomy (e.g., genus), and changes can be observed between samples and (3) functional capacity, which is a broad term that refers to the actions of microbes (such as metabolite production, or gene activity) through to the downstream effects and interaction with the host. This review primarily considers potential changes in measures of diversity and relative abundance in response to exercise.

Cross-sectional studies have reported that sedentary and active populations have different gut microbiota characteristics [8]. Both Barton et al. (2018) and Clarke et al. (2014) investigated the same group of participants using different analytical techniques with both finding that athletes had a greater diversity and functional capacity of gut microbiota compared to sedentary age-matched populations [8,9]. Furthermore, Castellanos et al. (2020) suggest that when transitioning from a sedentary lifestyle to an active lifestyle, there is a reduction in those bacteria related to disease coupled with an increase in taxa associated with health [10]. Collectively, the available findings suggest that engaging in exercise is related to a microbial profile in the gut that is associated with improved health outcomes. However, what is not well understood, is the potential relationship between exercise training and modifications to the gut microbiota in previously sedentary clinical populations. Furthermore, a previous systematic review suggested that vigorous exercise may elicit a greater change to the human gut microbiota compared to lower-intensity exercise [11]; however, it is not clear whether other aspects of exercise prescription (such as frequency, duration and type of exercise) may also impact the presence of and/or degree of changes.

The earliest systematic review examining a relationship between exercise and the microbiota that included some human data was published in 2019; only five of the 25 included studies reviewed in this paper included human data [12]. In 2020, a second systematic review was published, and this included 18 studies in humans, with half of those being cross-sectional in design [13]. More recent reviews have included cross-sectional studies (*n* = 25) [14], older adults only (*n* = 7) [15] or excluded those with clinical conditions and included cross-sectional studies (*n* = 38) [16]. While the early cross-sectional data have been invaluable for describing potential relationships between exercise and gut microbiota, these studies could not address the large interindividual variability in the composition of the gut microbiome in the same way as longitudinal studies have been able to do since. To avoid the potential loss of scope, the present systematic review aims to assess longitudinal data to determine how different exercise interventions potentially change the human gut microbiota. In addition, we consider whether changes in the microbiota differ between clinical and healthy cohorts. This review is aimed to inform future research, possible inform exercise prescription and improve the understanding of possible mechanisms and factors contributing to exercise and the human gut microbiota interactions.

## 2. Methods

This systematic review was conducted and is reported according to the Preferred Reporting Items for Systematic Reviews and Meta-Analyses (PRISMA) statement [17]. The review was registered with PROSPERO (CRD42022309854). This registration was amended following data collection. The meta-analysis was deemed to not be feasible due to the heterogeneity of alpha diversity reporting. Similarly, exercise prescription was found to vary dramatically between studies, and was considered a confounder when determining the effects of exercise on the gut microbiota in healthy and clinical populations. Due to this, exercise prescription in the reviewed literature was reported as a key aspect of this review. This was used to provide context as to the extent in which gut microbiota change when comparing populations may occur when considering exercise, as exercise prescription bias was significant.

On 21 February 2022, five databases were systematically examined by A.N.B: PubMed, Scopus, SportDiscus, CINAHL, and EMBASE. Searches were limited to full-text articles published in the English language in peer-reviewed journals. Key search terms included: ‘gastrointestinal microbiome’, ‘microbiome’, ‘microbiota’, ‘exercise’, ‘physical activity’, ‘adult’, ‘human’ and ‘NOT animal’ (for search terms used per database, please see Supplementary Table S1).

The inclusion criteria were: (i) design: randomised controlled trials, cohort studies and case-control studies; (ii) population: adults aged 18 years and older; (iii) intervention: any frequency, intensity, time or type of physical activity; (iv) control: comparison group receiving a different physical activity prescription, control group not receiving the intervention at any time point during the trial, waitlist control or crossover group or no comparison/control group; and (v) outcome: faecal analysis of microbiota diversity and/or microbiota taxonomy. Any study that also incorporated strategies that may have influenced the outcome (e.g., diet or inclusion of prebiotics or probiotics), where the effects of physical activity could not be isolated, was excluded.

Covidence was used for screening the titles and abstracts of articles identified through the search process. Duplicate removal was automated, and articles were screened by A.N.B. and R.E.W. to exclude those outside of the scope of the review. Following the screening, full-text articles were retrieved and independently assessed by A.N.B. and R.E.W. for eligibility according to the outlined inclusion criteria. Where discrepancies in article eligibility were identified, eligibility was discussed in the research group, with an independent assessment made by a third blinded arbiter if a consensus was not achieved (T.L.S.). Reference lists of eligible articles were examined to locate potential additional studies that met the inclusion criteria.

Study details, including the participant characteristics, exercise and control group prescriptions and outcome measures, were extracted independently by two authors (A.N.B. and R.E.W.). Where the assessment of microbiota diversity and/or microbiota taxonomy occurred at more than one time point during an intervention, the pre- and post-intervention outcomes were extracted, and other intra-intervention values were omitted. Participant characteristics and group classification were dependent on only one group; if a study included a group that was of clinical interest, this study was analysed with the perspective of this clinical group compared to the apparently healthy group included.

The methodological quality of included articles was independently assessed by A.N.B. and R.E.W. Randomised controlled trials meeting inclusion criteria were assessed for methodological quality using a six-item derivation of the nine-item Delphi list developed by Verhagen et al. Three of the nine Delphi criteria (blinding of the trainers, blinding of the outcome assessors and blinding of the participants) were deemed not to be appropriate for all types of included interventions. All criteria were equally rated using a ‘yes’ (1 point), ‘no’ or ‘unclear’ (0 points) answer format, with a quality score generated as a percentage of the maximum score for each included study. Non-controlled trials meeting the inclusion criteria were assessed using the Delphi, acknowledging that these would receive a lack of score for randomisation. Any discrepancies in methodological quality ratings were mediated by a third arbiter (T.L.S.). The results of the quality analysis can be found in Table 1 and Supplementary Table S2.

Results were analysed and reported using a combination of quantitative, descriptive and narrative data synthesis. All reported changes in the gut microbiota of studies included were collated with study and intervention characteristics by reports of increase, decrease or no reported change. Thereafter, summaries of reported changes in the gut microbiota across the included literature were produced (Supplementary Table S3, adapted from Ortiz-Alvarez et al. (2020)). The direction of change in microbiota diversity and relative abundance at multiple levels of taxonomy were tabulated corresponding to study characteristics. Subsequently, variables of interest (such as exercise intensity) were used to sort outcome measures and compare the number of studies reporting changes when considering this variable. No effect measures were suitable for this review, and a narrative synthesis of these findings was conducted. An issue when comparing the results of gut microbiome studies is that while changes are reported, the lack of changes in the abundance of specific taxa is rarely reported. We devised a system of reporting to account for this. Findings were organised by variables of interest (such as exercise intensity) and, unless stated in the text, net reported increases or decreases were reported as a proportion of studies finding this change (% of studies reporting change) compared to how many studies reported this variable (n_r_ = *x*) and how many studies (including those reporting change) likely were able to observe changes to this level but did not report a change within the bounds of the variable (n_total_ = *y*). For instance, the statement ‘increases seen in *Example bacterium* (50%, n_r_ = 4; n_total_ = 13)’ should be interpreted as, of the studies including this variable, two of the four studies (50%) reporting this level of taxonomy found an increase, and thirteen studies (including the four listed) potentially had the capacity to observe a change, but it was not reported, suggesting no change was reported. This approach was used unless the review is comparing the frequency of reporting a microbe in response to a single variable. In this case, the only number reported (n_r_) would be compared to the number that likely could assess this change (n_total_) within this variable, and this would only be reported once in each statement, e.g., *Example* was more commonly reported in *x* interventions (n_r_ = 4; n_total_ = 5) compared to *y* intervention (n_r_ = 1). Taxa that were reported in all studies likely to be capable of assessing this change in relation to the variable investigated were reported simply as (*n* = *x*).

## 3. Results

**Figure 1:**
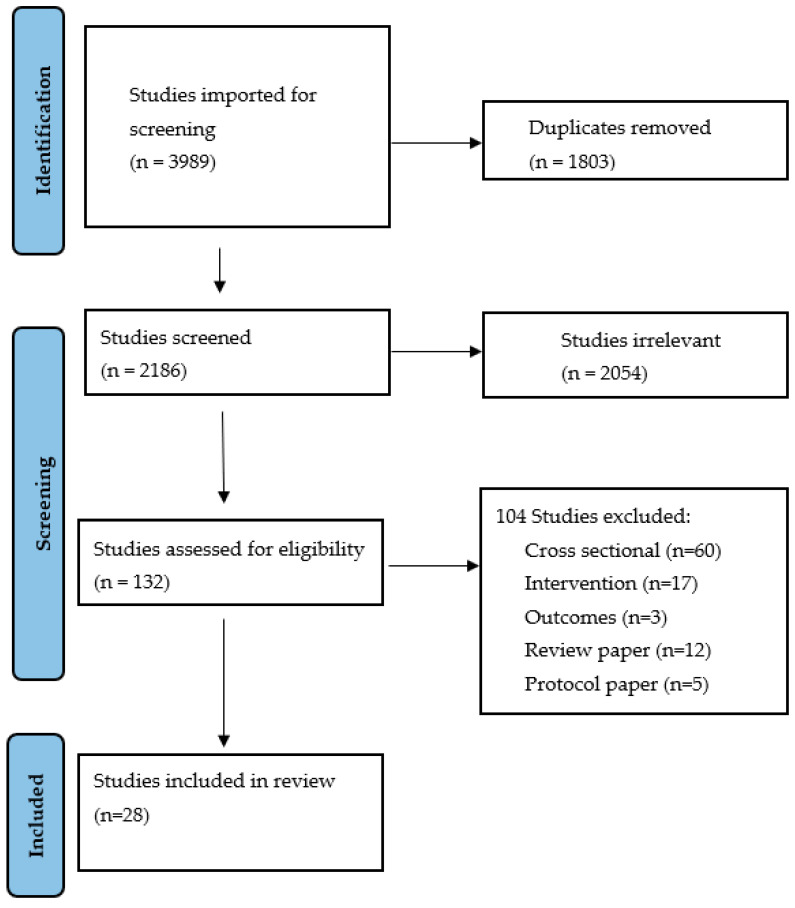
Study inclusion and screening (PRISMA).

Figure 1 shows that of the 3989 articles identified through the search, 28 of these were included in the qualitative synthesis. A total of 14 of these were comparator trials, with the remaining 14 single-arm interventions. Examples of studies similar to the selection criteria for this review that were ultimately excluded include studies that looked at the removal of activity (sedentary studies) [46], studies where there was no exercise only group or had a particular emphasis on diet in all groups [47,48], or that looked at microbes and metabolites external to the gut [49].

## 4. Participant Characteristics

Participant characteristics are shown in Table 2; 800 individuals participated across all studies (606 participants in the comparator trials and 194 in the single-arm trials). Of note, two studies were large randomised controlled trials; however, the effects of exercise were only observable in one of the groups in each study as the other arms had two interventions applied concurrently that could impact the gut microbiota, and there was no control for comparison. These studies have, therefore, been treated as single-arm trials for the purpose of this review [21,32]. Sixteen studies included participants from clinical populations. Of these, three studies compared data to an apparently healthy population [33,34,36], with the rest comparing exercise groups [35,40,41], waitlist or crossover [37,38,39,42], control groups [43,44,45] or did not include a comparison [30,31,32]. Of the remaining 12 ‘healthy population’ studies, four included apparently healthy sedentary and recreationally active participants [20,27,28,29]; one study was in a military setting [21], and seven studies assessed athletes only [18,19,22,23,24,25,26]. The mean age was 35.4 ± 13.1 years, and the mean BMI was 26.0 ± 3.8 kg/m^2^ (41.3 ± 12.7 years and 27.8 ± 3.38 kg/m^2^ for the clinical population studies, respectively; the mean age and BMI for the apparently healthy populations were 28.9 ± 10.6 years and 23.8 ± 2.7 kg/m^2^, respectively). Most clinical populations were characterised by metabolic disorders (*n* = 11, 60% of clinical studies) [30,31,32,34,36,37,38,40,41,43,44]. Other clinical population domains included neurological disorder (*n* = 1, 6%) [45], autoimmune (*n* = 1, 6%) [42], inflammatory bowel disease (*n* = 1, 6%) [39], myalgic encephalomyelitis/chronic fatigue syndrome (*n* = 1, 6%) [33] and elderly populations (*n* = 1, 6%) [35].

## 5. Intervention Characteristics

Details of the exercise interventions are summarised in Table 2. The majority of studies employed aerobic exercise interventions (*n* = 19) [18,19,20,21,22,24,26,29,31,33,34,35,36,37,38,40,41,42,44], while eight studies included a combination of resistance and aerobic exercise (labelled as concurrent training) [23,25,30,31,32,39,43,45] and a single study compared resistance to aerobic training [27]. Three studies were classified as acute with the exercise perturbation consisting of a single effort [19,22,33]. Another four studies were classified as short-duration interventions, equal to or shorter than three weeks in duration [21,26,36,41]. The remaining twenty-one studies employed longer exercise interventions, lasting from five to twenty-four weeks. Thirteen studies utilised low-to-moderate to moderate intensity exercise in at least one study arm [26,28,29,30,32,34,35,37,38,39,40,41,45], nine studies used moderate-to-high to high-intensity exercise [18,21,22,23,24,25,27,31,44] with two studies using maximal effort [19,33], four studies using variations of high-intensity interval training [20,36,42,43] and one used sprint interval training [41]. Two studies compared exercise intensity to selected outcomes [40,41]. Of the short-to-long intervention studies, participants exercised at least three times per week, while one study involved twice-weekly exercise training [42].

## 6. Outcome Measures

Stool samples were sequenced with 16S rRNA gene amplicon sequencing (*n* = 26), whole genome shotgun sequencing (*n* = 5) and one study [30] did not record their sequencing method. Details of sequencing methods and results of microbiota sequencing are provided in Table 3 and Supplementary Table S3. When reporting microbiota metrics and changes, twenty-one studies (75%) included a form of alpha diversity, twenty included beta-diversity assessment (71%) and eight (29%) reported at the level of phylum. Twenty (71%) studies reported at the level of genus; another seven reported on fewer than three genera, leaving thirteen (46%) studies reporting three genera or more; ten studies (36%) reported changes at the level of species. Twenty-six studies employed a form of dietary assessment during the intervention. Of these, three utilised diet control prior to sample collection. Another three studies asked participants not to change their eating habits for the duration of the study. Five studies did not control for nor assess diet during the intervention. Fifteen studies collected stool samples before and after their exercise intervention. Two additional studies included duplicate (consecutive) samples at baseline and either a follow-up sample post-intervention or duplicate samples post-intervention. The remainder of the studies collected three to four faecal samples throughout the intervention (*n* = 9) except for two studies, one of which collected fourteen samples per participant (every two weeks) and the other collected twenty-eight samples per participant (two samples per week)

## 7. Influence of Exercise Type on the Gut Microbiota

Concurrent exercise interventions (e., aerobic combined with resistance training) more commonly reported increases in measures of alpha diversity (50%, *n* = 6) compared to aerobic-only interventions (20%, *n* = 15). Beta diversity was reported to increase in 83% of concurrent trials (*n* = 6) compared to 42% of aerobic-only interventions (*n* = 12). Aerobic-only exercise interventions were the only interventions to report *Roseburia* and *Lachnospira*, where increases were reported in (50%, n_r_ = 6; n_total_ = 10) and (80%, n_r_ = 5; n_total_ = 10) of studies, respectively. *Dorea* abundance was reported to increase more often in response to aerobic interventions (n_r_ = 4; n_total_ = 7) compared to concurrent exercise interventions (*n* = 1). Similarly, aerobic interventions reported a higher abundance of *Ruminococcus* more commonly than concurrent interventions (n_r_ = 5; n_total_ = 9 compared to *n* = 1, respectively). Changes in *Bifidobacterium*, *Veillonella and Akkermansia* were similar between intervention types (aerobic interventions accounted for 60%, 50% and 67% of increased abundance in these genera, respectively).

## 8. Influence of Exercise Intensity on the Gut Microbiota

### 8.1. Low-to-Moderate and Moderate-Intensity Exercise

Thirteen studies used low-to-moderate or moderate-intensity exercise in at least one study arm [26,28,29,30,32,34,35,37,38,39,40,41,45]. 20% of studies examining changes in alpha diversity found an increase in alpha diversity (*n* = 10), while 45% of studies examining changes in beta diversity reported a higher diversity (*n* = 11). Two studies found an increase in *Bacteroides*, with another reported no change (n_r_ = 3; n_total_ = 6). A total of 50% of studies reporting *Faecalibacterium* changes used low-to-moderate intensity exercise (n_r_ = 4; n_total_ = 7). At the level of species, *Faecalibacterium prausnitzii* was not found to change in two studies, though a third did report an increase (n_r_ = 3; n_total_ = 6). This intensity of exercise included 66% of studies reporting an increase in *Prevotella copri* (n_r_ = 3; n_total_ = 6).

### 8.2. Moderate-to-High and High-Intensity Exercise

Nine studies used moderate-to-high and high-intensity exercise [18,21,22,23,24,25,27,31,44], and all reported to the level of genus. A total of 67% of studies reporting alpha diversity found a positive change (*n* = 6), while 75% of studies reporting beta diversity found a positive change (*n* = 4). At the level of phylum, consistent increases in *Firmicutes* were seen in all studies that reported this level of taxa (n_r_ = 3; n_total_ = 5). Similarly, decreases in *Bacteroidetes* abundance were found in studies reporting at this level (n_r_ = 2; n_total_ = 4). At the level of family, 67% of studies reporting improvements in *Lachnospiraceae* and *Ruminococceae* used moderate-to-high-intensity exercise (n_r_ = 3; n_total_ = 4). At the level of genus, moderate-to-high and high-intensity exercise included, most studies that reported a positive change in *Dorea* (60%, n_r_ = 5; n_total_ = 7), *Bifidobacterium* (60%, n_r_ = 5; n_total_ = 6), *Ruminococcus* (67%, n_r_ = 6; n_total_ = 7) and *Akkermansia* (67%, n_r_ = 3; n_total_ = 6) and 50% of studies reported improvements in *Veillonella* (n_r_ = 4; n_total_ = 5). The only two studies reporting an increase in *Streptococcus* utilised this exercise intensity (n_r_ = 2; n_total_ = 4). Two of three studies reporting *Roseburia* at this exercise intensity found a decreased relative abundance (n_r_ = 3; n_total_ = 4). Both studies reporting *Bacteroides* at this intensity reported a decreased abundance of this genus (n_r_ = 2; n_total_ = 3). Conflicting data were found for *Lachnospira* (n_r_ = 2; n_total_ = 4), *Faecalibacterium* (n_r_ = 2; n_total_ = 4), *Prevotella* (n_r_ = 2; n_total_ = 3) and *Collinsella* (n_r_ = 2; n_total_ = 4). At the level of species, both studies reported an increase in *Ruminococcus bicirculans* and utilised moderate-to-high intensity exercise (n_r_ = 2; n_total_ = 3).

### 8.3. High Intensity Interval Training (HIIT)

Four studies utilised HIIT [20,36,42,43]. No studies that prescribed HIIT reported a change in alpha diversity (*n* = 4), while three of the four studies reported an increase in beta diversity (*n* = 4). Three studies reported at the level of genus, and two studies of these reported an increase in *Bifidobacterium* (*n* = 2). There were conflicting data for *Roseburia*; one study reported increases in *Dorea*, *Ruminococcus* and *Veillonella abundance* and a decrease in *Prevotella*. None of the HIIT studies reported changes at the level of species.

### 8.4. Sprint Interval Training (SIT) and Maximal Effort

Three studies utilised SIT [41] or maximal effort interventions [19,33]. No studies reported changes in alpha diversity (*n* = 2) or beta diversity (*n* = 1) when using maximal effort or SIT protocols. One study utilising maximal intensity and the study utilising SIT found that *Lachnospira* abundance increased. Otherwise, no consistent changes were observed. One study that included maximal exercise efforts found increases in *Ruminococcus* and *Romboutsia*.

## 9. Influence of Exercise Frequency on the Gut Microbiota

Of those studies that used a training frequency of two to three sessions per week (*n* = 13), most did not find a change in alpha diversity; only one study reported an increase (9%, *n* = 11). A total of 58% of studies reporting beta diversity found an increase (*n* = 12). At the level of genus, studies that involved two to three training sessions per week included 50% of studies reporting increases in *Lachnospira* (n_r_ = 4; n_total_ = 10), 60% of studies reporting improvements in *Bifidobacterium* (n_r_ = 5; n_total_ = 9), 67% of studies reporting improvements in *Roseburia* (n_r_ = 3; n_total_ = 10) and both studies reporting improvements in *Oscillospira* (n_r_ = 2; n_total_ = 7).

Studies with a training frequency of four to five sessions per week (*n* = 5) reported an increase in alpha diversity in 33% of studies reporting this metric (*n* = 3) and 67% for those reporting changes in beta diversity (*n* = 3). No consistent changes were seen at the level of genus.

Studies with a training frequency of greater than five sessions per week (*n* = 4) found an increase in alpha diversity in 67% of studies reporting this change (*n* = 3). However, there was no change in beta diversity (*n* = 1). No consistent changes have been reported at the level of genus, though *Prevotella copri* was found to increase in two of the studies (50%, n_r_ = 4; n_total_ = 5).

Interventions that did not specify exercise frequency or involved less than one training session a week were not included in the frequency analysis (*n* = 6).

## 10. Influence of Intervention Duration on the Gut Microbiota

Studies that included an exercise intervention for 12 weeks or longer represented 50% of all reported improvements in alpha diversity. All those studies that assessed potential changes in beta diversity with a duration of six weeks or more (*n* = 13) reported improvements in beta diversity compared to shorter interventions of 0% (*n* = 4). At the level of genus, *Roseburia* decreased more commonly in interventions that lasted less than one week (*n* = 2), compared to a longer duration intervention (*n* = 1). All improvements in *Roseburia* occurred in response to interventions lasting more than four weeks (50%, n_r_ = 6; n_total_ = 10), while *Lachnospira* was reported to increase in studies lasting eight weeks or less (80%, n_r_ = 5; n_total_ = 10). *Dorea* was reported to increase in studies ranging from one week to twenty-six weeks (n_r_ = 5; n_total_ = 7), while increases in *Ruminococcus* were found both in studies lasting less than one week (*n* = 3) and also in those ranging from eight weeks to twenty-six weeks (*n* = 3) (n_r_ = 6; n_total_ = 10). Studies with an intervention duration of over nine weeks reported the majority of increases in *Veillonella* (75%, n_r_ = 4; n_total_ = 8) and *Bifidobacterium* (100%, n_r_ = 5; n_total_ = 9). Decreases in *Clostridium* were reported in studies with a duration of five weeks or less (100%, n_r_ = 3; n_total_ = 8). Changes in *Akkermansia* were inconsistent in relation to duration, with increases seen from one to twenty-six weeks (n_r_ = 3; n_total_ = 6).

## 11. Influence of Time Exercising per Session on the Gut Microbiota

Increases in alpha diversity were only found in studies that included more than 90 min of exercise per session; a total of 67% of these studies found an improvement in alpha diversity (*n* = 6).

Studies that involved 30 to 60 min of exercise per session most commonly reported improvements in *Lachnospira* (*n* = 3), with <30 min reporting an increase (*n* = 1) and >60 min reporting a decrease (*n* = 1). Increases in *Dorea* were only found in studies that included exercise lasting longer than 50 min (100%, n_r_ = 5; n_total_ = 7). Increases in *Ruminococcus* were also most commonly reported with exercise sessions lasting longer than 50 min (83%, n_r_ = 6; n_total_ = 10). Increases in *Streptococcus* were only reported in studies involving exercise sessions lasting more than 95 min (*n* = 2) (n_r_ = 2; n_total_ = 7). *Bacteroides* increased in exercise sessions ranging between 30 and 60 min (*n* = 2) and decreased in studies lasting more than 90 min (*n* = 2) (n_r_ = 5; n_total_ = 7). Increases in *Bifidobacterium* were most commonly reported in studies that included exercise sessions lasting between 30 and 60 min (80%, n_r_ = 5; n_total_ = 7). *Akkermansia* was more commonly found to increase in response to interventions that included 90 min exercise sessions (67%, n_r_ = 3; n_total_ = 4).

## 12. Response of the Gut Microbiota to Exercise in Healthy Compared to Clinical Populations

Of those studies that reported alpha diversity (*n* = 21), 18% of those in clinical populations reported an increase (*n* = 2), with the remainder finding no change (*n* = 9). In healthy populations, 40% of studies reported an increase in alpha diversity (*n* = 4), with the remainder finding no change (*n* = 6). Beta diversity (*n* = 17) was increased following exercise for 60% (*n* = 6) of clinical population studies that reported this metric (*n* = 10), with 57% (*n* = 4) reporting this change in healthy populations (*n* = 7).

Twenty studies reported a change at the genus level and increases in *Roseburia* and *Lachnospira* were more common in clinical populations compared to healthy populations (*n* = 2 vs. *n* = 1 (n_r_ = 6; n_total_ = 10), and *n* = 4 and *n* = 0 (n_r_ = 5; n_total_ = 10), respectively). Indeed, healthy populations reported decreases in *Roseburia* (*n* = 3). Increases in *Dorea*, *Ruminococcus*, *Romboutsia* and *Bifidobacterium* more commonly reported in healthy populations following exercise (*n* = 4 vs. *n* = 1, *n* = 5 vs. *n* = 1, *n* = 2 vs. *n* = 0, and *n* = 3 vs. *n* = 1, respectively). Healthy populations were more likely to show decreases in *Roseburia* (*n* = 3 vs. *n* = 0), *Prevotella* (*n* = 2 vs. *n* = 0) and *Bacteroides* (*n* = 2 vs. *n* = 0). Both healthy and clinical populations reported decreases in *Odoribacter* and *Clostridium*, as well as increases in *Veillonella* and *Akkermansia* with similar frequency.

Ten studies reported changes at the species level. These changes were more commonly reported in healthy populations (*n* = 6); two studies found that *Ruminococcus bicirculans* and *Prevotella copri* increased in healthy populations, and one study reported an increase in *Prevotella copri* in clinical populations. No other consistent changes in species were reported.

## 13. Discussion

The present systematic review investigated reported changes in the human gut microbiota in response to exercise interventions with the aim of better understanding the potential influences of exercise variables and disease status on changes to the gut microbiota. Analyses included 28 studies with a variety of populations (clinical and healthy), exercise interventions, sequencing techniques and targets of sequencing. Exercise interventions were assessed by exercise type, intensity, frequency, and time, as well as the duration of the training intervention. Outcomes that were based on the population were also investigated; clinical and apparently healthy populations were compared in a number of studies. Clinical populations included metabolic conditions, neurological disorders, autoimmune disorders, myalgic encephalomyelitis and elderly populations. Healthy populations included apparently healthy, recreational and elite athletes and military populations. The findings suggest that differences in exercise delivery influence changes in the gut microbiota in response to training. While population variations were found to produce a similar response at the level of diversity, genus responses were found to vary.

### 13.1. Exercise Intervention Characteristics

Concurrent exercise (e.g., aerobic, combined with resistance training) appeared to influence alpha and beta diversity more than aerobic-only interventions. Aerobic-only exercise interventions reported more common changes at the level of genera (*Roseburia*, *Lachnospira*, *Dorea* and *Ruminococcus*, in particular). Exercise type was only directly assessed in one randomised controlled trial [27]. This study found that aerobic exercise changed the microbiota within two weeks of exercise and established a stable new composition between six and eight weeks [27]. No changes were seen with resistance training alone [27]. It appears that most of the changes observed in the gut microbiota with exercise can likely be attributed to aerobic exercise, with resistance training having only a small influence, that may compound with aerobic exercise. Acknowledging this, previous research has indicated that sport type, as well as diet, influences the gut microbiota profiles of athletes [48]. However, when controlling factors in the diet to assess the influence of sport type, there were still differences in the gut microbiota profiles observed between bodybuilders, runners and controls (healthy sedentary men) [48]. Further research is needed to determine whether the high specificity, history of training and volume of resistance training commonly seen in bodybuilding may impact the gut microbiota, of which none of the studies in this review included to this degree. Furthermore, evidence suggests that a gut-muscle axis may exist, further implicating resistance training and muscle mass interactions with the gut [50,51].

Studies using high-intensity exercises were more likely to report improvements in alpha diversity, beta diversity, phylum abundance and family abundance. At the level of genus, these studies also more commonly report changes when compared to those studies that used low-to-moderate intensity exercise, single maximal efforts and SIT interventions, although the sample size was low for the latter two intensities of exercise delivery. When comparing outcomes from high-intensity and HIIT studies, changes in beta diversity and genera are relatively consistent; high intensity exercise creates a disturbance to the gut resulting in changes in the microbiota that may be associated with improved health. This finding agrees with the previous reviews that have assessed cross-sectional data, in which it was found that higher-intensity exercise was linked to unique microbiota profiles [11]. Interventions that reported decreases in some commensal bacteria and increases in potentially harmful bacteria also involved high-intensity exercise, suggesting that high-intensity exercise, especially for a long duration per exercise session, may elicit perturbations that could, at least acutely, be deleterious to health. However, there is currently insufficient evidence to conclude that there are potentially long-term health consequences resulting from acute changes in what may be considered harmful bacteria in response to high-intensity exercise.

It appears that while exercising two to three times per week is sufficient to influence beta diversity and abundance at the level of genus, four to five weekly sessions are needed to elicit some change in alpha diversity (33%)—and more than five sessions a week are needed for more consistent increases (67%). Again, the influence of diet on those changes reported in response to exercise interventions is not well understood. As very high levels of physical activity are often accompanied by an increase in energy intake, future research needs to control for diet when assessing potential changes in alpha diversity with exercise training. Additionally, relatively few studies have included more than three and five exercise sessions a week (*n* = 5 and *n* = 4, respectively) compared to those that have used two to three sessions a week (*n* = 13). This may explain why some genera were not consistently reported in higher frequency interventions; six studies did not report exercise frequency, and these were not included in the analysis. It is unclear whether these changes may have been observed in other studies had different analysis techniques been utilised.

Exercise interventions lasting more than four weeks were responsible for most of the reported changes at the genus level. Changes in diversity metrics were found at six weeks (beta diversity) and 12 weeks (alpha diversity). The longer duration (greater than eight weeks) exercise interventions were more likely to find changes in the microbiota at the genus level. In noting this, interventions lasting less than four weeks reported changes in *Roseburia* (decrease), *Ruminococcus* (increase) and *Clostridium* (decrease). These changes are likely to be compounded by the maximal intensity or very high exercise session durations used in these studies. Of interest, *Lachnospira* appeared to increase in response to eight weeks of training, after which there was no further change. It is unclear as to whether this suggests that the perturbation of exercise only influences this genus in the short term. *Veillonella* is one of the most highly discussed genera in the context of exercise and microbiota interactions, with Scheiman et al. (2019) identifying its potential role in endurance exercise as a metaboliser of lactate [52]. In agreement with Scheiman et al. (2019), who researched experienced cyclists with varying durations of training per week, this review found that longer duration interventions were likely to report *Veillonella* increases, suggesting that repeated and prolonged exercise behaviour may be linked to this genus [52]. It appears that this genus may also respond acutely to exercise, with Grosicki et al. (2019) reporting a large increase in the abundance of *Veillonella* following an ultra-marathon. *Veillonella* was also shown to increase in exercise studies with clinical populations, potentially supporting Scheiman’s suggestion that *Veillonela* can increase based on exercise demands. However, the same can be said for other genera assessed in this review. Based on the relative interest in *Veillonella* and the mixed findings, with some studies finding significant changes and others finding no change, it is possible that those individuals showing an increase in *Veillonella* have a significant presence of this microbe prior to training.

The duration of exercise sessions was highly predictive of change in the gut microbiome diversity, with sessions lasting more than 90 min producing the most consistent change in alpha diversity. Thirty minutes appeared sufficient to produce increases in *Lachnospira* and *Bacteroides;* however, longer sessions appeared to blunt this increase or even result in decreases. Similarly, 50 min per session was associated with increases in *Dorea*, *Bifidobacterium* and *Ruminococcus* with decreases in *Ruminococcus* reported in studies where training sessions lasted longer than 90 min. These longer duration sessions (>95 min) also appear to be related to increases in *Streptococcus.* While alone this is not concerning, at the level of genus it may be a potential tool to further investigate the environmental changes that may be linked to microbiota change with exercise, authors of both articles reporting an increase in *Streptococcus* comment that this is a genus that does contain pathogenic species [21,23]. *Akkermansia* was also reported to increase in these longer duration sessions; *Akkermansia* is a commonly reported genus that has a higher abundance in active individuals [9,53]. This review found a relatively high agreement between reporting and capacity to investigate this species, with 50% of those likely to have the capacity to see a change reporting an increase with exercise. The implications of this are not clear, as there is some evidence to suggest that it may be related to inflammation [54] and obesity [55]. Given these findings, it appears time per session may induce a dose-dependent perturbation to the gut microbiota with genera differentially influenced. This may suggest that mechanisms relating exercise to microbiota are linked to the duration of a change in homeostasis or perturbation, as has been previously suggested [11].

It appears that aerobic training at higher intensities is most likely to elicit changes to the gut microbiota, particularly changes in alpha diversity. There appears to be a dose–response, with a possible ‘inverted U’ describing the relationship; low frequency and duration of exercise results in little to no change, 30–90-min sessions 3–5 times per week produce most of the change, and exercise lasting longer than 90 min and more than five times per week produces some change but a higher likelihood of a potentially negative change to the gut [23]. Indeed, it appears that both the exercise duration and intensity may be key variables that influence acute changes to the gut, with the evidence here providing a preliminary suggestion of 150–270 min of moderate to high-intensity exercise required per week to modify the gut microbiota. A short review by Keirns et al. (2016) encompasses many of the potential roles and mechanisms by which exercise interactions with the gut may follow the model of hormesis [56]. An increase in intestinal permeability is believed to influence how the gut microbiota, metabolites and host interact due to greater contact with microbes, metabolite sharing and nutrient availability. In isolation, this has been considered negative to health; however, in the context of exercise, it may be necessary to promote positive adaptation. Highlighted by Keirns et al. (2016), an increase in intestinal permeability of ~241% with 20 min of high-intensity exercise (≥80% HRmax) was found by Marchbank et al. in 2011 [57]. Interestingly, a subsequent study by Zuhl et al. (2014) found that moderate-intensity exercise (65% HRmax) for 60 min or more resulted in a ~277% increase in this same marker [58]. These findings suggest a mechanism that aligns with and may explain the findings of this review, with 20 min of high intensity exercise producing similar changes to 60 min of moderate intensity exercise. These findings suggest that possibly, intensity and duration of exercise can be manipulated to promote gut microbiota change. Similarly, changes in core temperature [59] and changes in splanchnic blood flow (hypoperfusion and reperfusion) [60,61], among other mechanisms, have been linked to changes in gut permeability. This suggests that the mechanisms, or part of the mechanism, linking exercise to gut microbiota change can be promoted via both high-intensity exercise and longer-duration exercise separately.

The available evidence suggests that a certain level of disruption or hormesis is necessary to influence changes in the gut microbiota of humans; however, there may be a ceiling effect, as seen with the plateaus in microbiota change in some studies in this review. To this point, an exercise intervention duration of eight weeks or more appears to produce the most consistent change across alpha diversity, beta diversity and most genera. Furthermore, the majority of studies collected samples only before and immediately after the exercise intervention, and the time course of changes is not clear. Of the two studies that sampled faecal matter during the exercise intervention, the limited available data suggest that most of the observed changes in the microbiota may occur within two weeks of training before plateauing [27]. In the case-control study, two elite athletes experienced fluctuations in diversity; however, peaks were seen at the time of high training volume and competition, with large diversity changes occurring within four weeks [25]. These studies suggest that adaptation to exercise occurs rapidly; however, further research is required to verify these findings.

Based on the changes observed in this review, the removal of the exercise stimulus reverses changes in as little as three weeks in previously sedentary individuals [27]. This is shorter than the duration found previously, in which there was a reversal of microbiota and function after six weeks of washout (no physical activity) for both lean and obese participants [34]. A similar trend was found in a study with patients who had celiac disease [42]. One of these studies included an assessment following reduced exercise load was in competitive runners, which found no significant change in microbiota profiles following a taper [18]. Another study found similar results in a smaller sample size, with a partial reduction seen after three months [24]. These findings suggest long-term exercise behaviour may be required to maintain the microbiota changes in previously inactive individuals but may not be as important in active individuals. It may also be the case that a change in exercise volume has less of an impact on active individuals, as they may still be conducting exercise rather than reverting to sedentary behaviour. What is not clear is how long exercise adherence is required to establish a unique homeostasis that is resilient to the removal of exercise. It has been suggested that creep in microbiota profile from any perturbation will result in a permanent change to homeostasis with sufficient time, and indeed cross-sectional literature does suggest that this may occur with exercise as active individuals and athletes more commonly present the characteristics of an ‘active’ microbiota seen with exercise in this review when compared to sedentary individuals [8,62]. Studies that include exercise (training) tapering, maintenance, or follow-up measures after exercise completion will provide valuable insight.

### 13.2. Population Influence

Many clinical populations present with gut microbiota dysbiosis or different microbial composition compared to healthy individuals [4,63,64]. This review considered collective findings from varied populations to address whether changes in the gut microbiota in response to exercise training differed with health status. Acknowledging the variety of clinical conditions included in this review, there were some varied responses between groups, yet some changes were similar to those with healthy populations. In particular, it appears that beta diversity changes in response to exercise were similar in both clinical and healthy populations. However, in response to exercise, alpha diversity was more likely to change in healthy populations compared to clinical populations. Given that those studies using healthy populations also used higher intensities of exercise, it remains to be determined whether the difference in alpha diversity across populations was more due to exercise prescription rather than health status. Additionally, it appears feasible that immune implications and/or medications used in clinical populations may interact with the gut microbiota and possibly blunt some changes [65]. At the level of genus, four genera responded similarly between populations, while six responded differently. Again, whether this is related to health status, baseline microbiota composition or, for example, the intensity of exercise training is yet to be determined. Interestingly *Roseburia* and, to some extent, *Lachnospira*, did increase with exercise more commonly in clinical populations, with these genera commonly seen to be reduced in clinical populations at baseline compared to healthy populations and are linked with potential benefits to health [66,67]. Exercise may then help to upregulate genera commonly considered to be contributory to health through SCFA (particularly butyrate) production [68,69,70]. Regardless, it is important to note that across both healthy and clinical populations, the genera most commonly reported to change in response to exercise training appear to be those significantly involved in SCFA production [16].

### 13.3. Limitations

The primary limitation of this review is that results were only included as they were reported in each study. Given that the reporting increase, decrease and no change across every level of taxonomy for each study is not feasible, this means that many of these studies likely found no change in microbes with no need to report this, especially those using metagenomic analysis. An example of this is *Bacteroides*, which is a highly discussed and observed genus in the wider human gut microbiota literature; however, this review found only three studies reported this genus. It is, therefore, likely that the one study reporting no change was supported by an unknown number of other studies. As such, the results of this review should be interpreted as exercise likely having either no effect or a positive effect (in the case of *Bacteroides*) as per each change reported.

The current review is also limited by the high heterogeneity of studies assessed. This has been the nature of microbiota research as an emerging field in exercise and in humans; however, future research will benefit from standardisation of some of the techniques from stool sample collection to downstream analysis. A recent study highlighted that the most popular and currently feasible way of analysing the gut microbiota (compositional approach) may underestimate the changes made in the gut over time due to the imposed constraints of abundance analysis [71]. An alternative is not clear, and a consensus on the best approach is yet to be reached; however, being aware of this change in interpreting research will be important for future studies. Further research is required to verify how different techniques may impact the interpretation of these outcomes. In the immediate future, it is recommended that high-sensitivity approaches of both composition and function, such as metagenomic analysis, are used when possible. A key article by Shanahan and colleagues (2021) highlights this capacity with the emphasis that there are multiple states of a “healthy” microbiota and that the detail down to strains of bacteria can be important for interpreting these outcomes should clinical relevance be a goal of research or application [72].

Control standards varied considerably between studies, primarily concerning diet analysis, repeated sample collection and frequency of sample collection. Again, there is no consensus as to what ideal standards should be, and as the literature stands, much can be interpreted from the collection of studies presented in this review. However, for the strength of individual studies, understanding the intra-individual variation and influence of diet on stool samples and the interpreted microbiota will strengthen the conclusions drawn from these studies.

To support the intraindividual variation and its role in microbiota research in humans, a growing body of literature suggests that case-wise/intrapersonal assessment in a functional capacity may be a positive way to understand microbiota changes [72] and to acknowledge that the abundance of metabolic pathways is more consistent across people than taxonomic composition [73] and perhaps this will provide more translatable outcomes in microbiota and microbiome research in humans. To reflect this, more frequent samples and duplicates during an intervention enhance both the understanding of the time course of change as well as intraindividual variation, respectively. Combining these standards of assessment may also allow a greater appreciation for how the exercise–microbiota interaction varies between individuals and provide more insight into personalised medicine and exercise prescription. Indeed, baseline characteristics of the human gut microbiota have been associated with cardiorespiratory fitness and perhaps may be an avenue to provide insight into a response to exercise [27,34,74]. It is possible this may also be related to the difference in gut microbiota response between clinical and healthy populations.

Finally, this review did not assess the data recorded in repositories and, as such, all results were drawn directly from the body of articles or supplementary files where possible. There is a risk of under- or over-reporting some findings in this review due to the analysis techniques used, and perhaps a review investigating repository data, such as the work by Bisanz et al. (2019), in high fat diets in murine models may be warranted [75]. Similarly, there was some bias in exercise prescription in the studies assessed. The current review found that high-intensity exercise influenced multiple metrics of the microbiota; however, of the nine studies included, only two involved clinical populations. However, with moderate-intensity exercise interventions, most studies investigated clinical populations. Further RCTs investigating variations in exercise principles are required in both populations to assess these changes. Practically, in the case of future RCTs, waitlist or crossover control structures would improve the ability to interpret changes to the gut microbiota in response to exercise training. Mechanistic studies will also be crucial to enhance our understanding of the role of gut microbiota and health [76,77]. The challenges discussed here are not unique to the exercise microbiota literature; with much of microbiota research striving to optimise translational potential [78], the research strategies will continue to be modified and optimised to benefit health and our understanding of the interactions between the microbiota and humans. Key findings and recommendations from the current body of literature can be seen in Table 4.

## 14. Conclusions

This is the first systematic review to assess only longitudinal study designs and varied populations when considering the interaction between exercise and the human gut microbiota. We were able to compare exercise dose through frequency, time, type and intensity of exercise across 28 studies. While single bouts of high-volume exercise have been shown to influence the gut microbiota, it was found that moderate-high, but especially high-intensity interventions, for more than 30 min, three or more times per week and for more than eight weeks resulted in the most consistent changes on the human gut microbiota. When potential differences between clinical and healthy populations were compared, microbiota changes were observed in both populations with exercise; however, specific genera that responded to exercise appeared to differ. Further research is required to verify these findings, and future studies would benefit from waitlist or crossover controls to account for inter and intraindividual variability in the gut microbiota. An improved understanding of how different types of exercise affect the human gut microbiota of different populations will inform the development of the most appropriate interventions to improve health via the gut microbiota and assist in chronic disease prevention and management.

## Figures and Tables

**Table 1:** Study design and participant characteristics.

Reference	Study Design	Study Quality	Sample Size	Groups, Male/Female (%)	Age (Years)	BMI (kg/m^2^)	Condition/ Intervention Group	Control Group	Classification	Microbiota Change (Diversity and Relative Abundance of Genera)
Craven et al., 2021 [18]	Single-arm	66.7%	14	Male 57%	F: 22 ± 3.4 M: 20.7 ± 3.2	F: 20.0 M: 21.43 (calculated)	Middle-distance runners (competitive)	NA	Athlete	Alpha-diversity—NR Beta-diversity—NR ↓ *Haemophilus*
Tabone et al., 2021 [19]	Single-arm	50%	40	Male 100%	35.79 ± 8.01	22.75 ± 2.12	Cross-country runners (elite)	NA	Athletes	~Alpha-diversity ~Beta-diversity ↑ *Blautia*, *Ruminococcus*, *Romboutsia* ↓ *Clostridium*
Zeppa et al., 2021 [20]	Single-arm	66.7%	18	Male 100%	22 ± 2	22.3 ± 2.7	Healthy (sedentary)	NA	Healthy	~Alpha-diversity ↑ Beta-diversity ↑ *Dorea*, *Ruminoccus*, *Bifidobacterium* ↓ *Roseburia*, *Prevotella*
Karl et al., 2017 [21]	Randomised Controlled Trial (Single-arm for exercise)	83.3%	18	NR	19 ± 2	23.6 ± 1.8	Healthy (military)	No: 73 participants in remainder of study	Military	↑ Alpha-diversity Beta-diversity—NR ↑ *Dorea*, *Ruminococcus*, *Streptococcus*,*Paraprevotella*, *Eggerthella*, *Akkermansia*, *Fusobacterium* ↓ *Roseburia*, *Lachnospira*, *Blautia*, *Blautia*, *Faecalibacterium*, *Odoribacter*, *Bacteroides*, *Collinsella*
Zhao et al., 2018 [22]	Observational	66.7%	20	Male 80%	31.6 ± 6.1	22.6 ± 2.1	Runners (amateur)	NA	Athletes	~Alpha-diversity Beta-diversity—NR ↑ *Ruminiclostridium*, *Coprococcus*, *Pseudobutyrivibrio*, *Ruminococcus*, *Romboutsia*, *Mitsuokella*, *Collinsella*, *Actinobacilus* ↓ *Roseburia*
Grosicki et al., 2019 [23]	Observational	50%	1	Male 100%	32	22.14	Ultra-marathon runner	NA	Athletes	↑ Alpha-diversity Beta diversity—NR ↑ *Faecalibacterium*, *Streptococcus*, *Veillonella*, *Haemophilus* ↓ *Subdoligranulum*, *Alloprevotella*
Keohane et al., 2019 [24]	Observational	50%	4	Male 100%	25.5 ± 1.3	24.4 ± 1.4	Endurance rowers	NA	Athletes	↑ Alpha-diversity Beta-diversity—NR ↑ *Roseburia*, *Dorea*, *Subdolilogranulum*, *Prevotella* ↓ *Bacteroides*
Barton et al., 2020 * [25]	Observational	83.3%	2	Male 100%	31.5 (30–33)	30.2 (28.6–31.7)	Marathon participant and triathlete	NA	Athlete	↑ Alpha-diversity ↑ Beta-diversity ↑ *Veillonella*, *Akkermansia*, *Bifidobacterium*
Oliveira et al., 2022 [26]	Observational	66.7%	17	Male 0%	24.1 ± 3.4	21.4 ± 1.7	Athletes (elite)	NA	Athletes	~Alpha-diversity ~Beta-diversity
Bycura et al., 2021 [27]	Controlled trial	83.3%	56	AT: 28 Male 25% RT: 28 Male 46%	AT: 20.54 ± 1.93 RT: 21.28 ± 3.85	AT: 24.41 ± 4.20 RT: 23.77 ± 4.15	Healthy young adults (Aerobic)	Healthy young adults (Resistance)	Healthy	Alpha-diveristy—NR AT: ↑ Beta-diversity
Erlandson et al., 2021 * [28]	Controlled trial	83.3%	22	Male 95%	58 (55, 63.8)	27.4 (24.6, 31)	Healthy (sedentary) High-intensity exercise	Healthy (sedentary) Moderate intensity exercise	Healthy	~ Alpha-diversity ↑ Beta-diversity ↑ *Oscillospira*, *Bifidobacterium*, *Succinivibria* ↓ *Prevotella*, *Oribacter*
Resende et al., 2021 [29]	Randomised Controlled Trial	100%	28	I: 14 Male 100% C: 14 Male 100%	I: 25.58 ± 5.07 C: 25.5 ± 4.66	I: 25.28 ± 4.11 C: 23.68 ± 3.29	Healthy (sedentary)	Healthy (sedentary, no exercise intervention)	Healthy	~Alpha-diversity ~Beta-diversity
Huber et al., 2019 * [30]	Single-arm	66.7%	44	Male 65.9%	41 (24–61)	31.3 (27.3,34)	NAFLD	NA	Clinical	Alpha-diversity—NR ↑ Beta-diversity Abundance—NR
Verheggen et al., 2021 [31]	Single-arm	66.7%	14	Male 50%	51 ± 11	34.9 ± 4.9	Obese (sedentary)	NA	Clinical	~Alpha-diversity ~Beta-diversity ↑ *Lachnospira*, *Ruminococcus*
Cronin et al., 2018 * [32]	Randomised Controlled Trial (Single-arm for exercise)	100%	30	Male 44%	35 (28, 38)	27.9 (25.1, 29.2)	Obese (sedentary)	No: 90 participants in remainder of study	Clinical	↑ Alpha-diversity ↑ Beta-diversty
Shukla et al., 2015 [33]	Observational	66.7%	20	ME/CFS: 10 Male 20% C: 10 Male 20%	ME: 48.6 ± 10.5 C: 46.5 ± 13	ME: 23.9 ± 4.3 C: 24.6 ± 3.3	Myalgic Encephalomyelitis/Chronic Fatigue Syndrome	Healthy Control	Clinical	Alpha-diversity—NR Beta-diversity—NR ↑ *Lachnospira*
Allen et al., 2018 [34]	Controlled trial	83.3%	32	L: Male 50% O: Male 21%	L: 25.1 ± 6.52 O: 31.14 ± 8.57	L: 22.21 ± 2.76 O: 35.71 ± 5.11	Obese (sedentary)	Lean (sedentary)	Clinical	~Alpha-diversity ↑ Beta-diversity O: ↑ *Lachnospira*, *Bacteroides*, *Collinsella* ↓ *Faecalibacterium* L: ↑ *Lachnospira*, *Faecalibacterium*, “Butyrate producers” ↓ *Bacteroides*
Morita et al., 2019 * [35]	Controlled trial	100%	32	I: 15 Male 100% C: 14 Male 100%	I: 70 (66–75) C: 70 (66–77)	I: 21.7 (18.9–23.1) C: 20.6 (18.7–24)	Elderly women (sedentary) Aerobic exercise	Elderly women (Sedentary) Trunk exercise	Clinical	Alpha-diveristy—NR Beta-diversity—NR ↑ *Bacteoides* ↓ *Closdrium*
Rettedal et al., 2020 [36]	Controlled trial	83.3%	32	I: 15 Male 100% C: 14 Male 100%	20–45	I: 29.6 ± 2.7 C: 22.7 ± 2.1	Overweight men (sedentary)	Lean men	Clinical	~Alpha-diversity ~Beta-diversity I: ↓ *Subdoligranulum* C: ↑ *Subdoligranulum*
Taniguchi et al., 2018 [37]	Randomised Controlled Trial	83.3%	33	I: 15 Male 100% C: 17 Male 100%	62–76	I: 22.9 ± 2.5 C: 22.9 ± 2.5	Diabetic (T2D) and pre diabetic	Diabetic (T2D) and prediabetic (Crossover)	Clinical	~Alpha-diversity ~Beta-diversity ↑ *Oscilllospira* ↓ *Clostridium*
Munukka et al., 2018 [38]	Randomised Controlled Trial	83.3%	22	Male 0%	36.8 ± 3.9	31.8 ± 4.4	Overweight (sedentary)	Overweight (sedentary) (Waitlist)	Clinical	~Alpha-diversity ↑ Beta-diversity ↑ *Dorea*, *Akkermansia* ↓ *Odoribacter*
Cronin et al., 2019 * [39]	Randomised Controlled Trial	83.3%	17	I: 8 Male 68.2% C: 9 Male 85.7%	I: 33 (31,36) C: 31 (31,36)	I: 28.1 (26.2, 32.4) C: 27.2 (24.5, 33.7)	Inflammatory bowel disease (Chron’s and UC)	Inflammatory bowel disease (crossover)	Clinical	~Alpha-diversity ~beta-diversity
Kern et al., 2020 * [40]	Randomised Controlled Trial	66.7%	130	Bike: 19 Male 42% Mod: 31 Male 55% Vig: 24 Male 50% C: 14 Male 57%	Bike: 35 (28, 43) Mod: 33 (27, 38) Vig: 39 (33, 42) C: 38 (30, 42)	Bike: 30.0 (28.3, 33.9) Mod: 29.3 (27.4, 30.5) Vig: 29.9 (28.2, 32.1) C: 29.9 (27.6, 32.3)	Overweight/obesity (sedentary) (Exercise Intensity x 3 groups)	Overweight/obesity (sedentary) (Usual care)	Clinical	↑ Alpha-diversity ~Beta-diversity
Motiani et al., 2020 [41]	Randomised Controlled Trial	66.7%	26	SIT: 13 MICT: 13 Male 61%	40–55	NR	Diabetic (T2D) and prediabetic (sedentary) SIT	Diabetic (T2D) and prediabetic (sedentary) MICT	Clinical	~Alpha-diversity Beta-diversity—NR SIT: ↑ *Lachnospira* ↓ *Blautia*, *Clostridium* MICT: ↑ *Faecalibacterium*, *Veillonella* ↓ *Blautia*, *Clostridium*
Warbeck et al., 2020 [42]	Randomised Controlled Trial	100%	41	I: 20 Male 20% C: 21 Male 10%	I: 42 ± 12.3 C: 36.2 ± 10.2	I: 27.0 ± 5.2 C: 28.7 ± 6.1	Celiac (sedentary)	Celiac (sedentary) waitlist	Clinical	~Alpha-diversity ↑ Beta-diversity I: ↑ *Roseburia*, *Adlercretzia* C (waitlist): ↑ *Veillonella*, *Bifidobacterium*
Dupuit et al., 2021 [43]	Randomised Controlled Trial	100%	29	I: 14 Male 0% C: 15 Male 0%	I: 58.8 ± 5.3 C: 60.9 ± 4.8	I: 30.3 ± 3.5 C: 31.5 ± 3.4	Post-menopausal women with overweight or obesity (sedentary)	Post-menopausal women with overweight or obesity (sedentary, no intervention)	Clinical	~Alpha-diversity ↑ Beta-diversity
Mahdieh et al., 2021 [44]	Randomised Controlled Trial (pilot study)	83.3%	18	I: 9 Male 0% C: 9 Male 0%	I: 23.87 ± 3.13 C: 26.37 ± 1.68	I: 27.76 ± 1.60 C: 28.41 ± 2.81	Overweight Women	Overweight Women (no exercise intervention)	Clinical	Alpha-diversity—NR Beta-diversity—NR I: ↑ *Lactobacillus*, *Bifidobacterium* C: ↑ *Lactobacillus*
Mokhtarzade et al., 2021 [45]	Randomised Controlled Trial	83.3%	42	I: 21 Male 0% C: 21 Male 0%	I: 35.06 ± 8.18 C: 36.38 ± 9.13	I: 23.47 ± 2.61 C: 22.62 ± 2.00	Multiple Sclerosis	Multiple Sclerosis (no exercise intervention)	Clinical	Alpha-diversity—NR Beta-diveristy—NR I: ↑ *Prevotella* ~*Bacteroides*

Data are mean ± SD unless otherwise stated, * Mean (total range reported); I = Intervention, C = Comparator, BMI = Body mass index, NAFLD = Non-alcoholic fatty liver disease, NA = Not available, F = Female, M = Male, NR = Not reported, ME/CFS = Myalgic encephalomyelitis/chronic fatigue syndrome, L = lean, O: Obese, AT = Aerobic training, RT = Resistance training, HI = High-intensity, Mod = Moderate intensity, Vig = Vigorous intensity, SIT = Sprint interval training, MICT = Moderate intensity continuous training, T2D = Type two diabetes mellitus, UC = Ulcerative Colitis.

**Table 2:** Details of exercise interventions.

Reference	Dropout Rate (%)	Aerobic/Resistance	Type	Duration of Intervention	Intensity	Time per Session	Frequency per Week	Adherence
Craven et al., 2021 [18]	NR	Aerobic	Running	7 weeks	Reporte as volume: 3 weeks of normal training, 3 weeks of high-volume training (+30% training volume), one week taper	NR	Prescribed per participant	NR
Tabone et al., 2021 [19]	0%	Aerobic	Treadmill and running	NA	Maximal intensity	Treadmill: until volitional fatigue Track: max pace 1 km	Single effort	100%
Zeppa et al., 2021 [20]	5.50%	Aerobic	Cycle ergometer	9 weeks	HIIT mixed with LIT (each session had HI at 20% of session)	55 min, 60 min, 70 min (3 weeks each)	3× 55 min first 3 weeks, 4× 60 min 3 Weeks, 5× 70 min for last 3 weeks	NR
Karl et al., 2017 [21]	0%	Aerobic	Cross-country ski/march	4 days	50:10 min work:rest	NR (51 km total distance)	NA	100%
Zhao et al., 2018 [22]	0%	Aerobic	Running	Single effort	Moderate to vigorous intensity	92–160 min	NA	100%
Grosicki et al., 2019 [23]	0%	Aerobic (>80%) Resistance (<20%)	Running “Strength”	23 weeks	Moderate to high	~666 min per week	115–124 km per week	100%
Keohane et al., 2019 [24]	0%	Aerobic	Rowing	33 days, 22 h	Moderate to high	2 h increments, totalling 349.9 h each	Average: 151.8 km/day (12 h)	100%
Barton et al., 2020 [25]	0%	Aerobic (*n* = 1) Concurrent (*n* = 1)	Sport specific	26 weeks	NR	1–8 h	NR	NR
Oliveira et al., 2022 [26]	NA	Sport specific	Sport specific	3 days	3–6 RPE	666 min	10 sessions over 3 days	NR
Bycura et al., 2021 [27]	0%	AT: aerobic RT: resistance	AT: 2× group cycling sessions + 1× rotating CRE Activity RT: 3–6 sets of 6 12 reps full body exercise	8 weeks	AT: 60–90% HRmax RT: 70–85% 1RM	60 min	3 sessions	100%
Erlandson et al., 2021 [28]	32%	Concurrent	Treadmill Four weight based exercises	24 weeks	Periodised RT and AT until week 12 then randomised to moderate (40–50% VO2 max and 60–70% 1RM) or high intensity (60–70% VO2max and >80% 1RM) with same intervention structure	20 min to 50 min AT, 4 exercises, 3 sets 8 reps	3 sessions	NR
Resende et al., 2021 [29]	14%	Aerobic	Cycle ergometer	10 weeks	Moderate intensity (steady state weeks 1 and 2, 65% VO2 progressive load weekly for weeks 3–10)	50 min	3	100% compliance
Huber et al., 2019 [30]	6.80%	Web based concurrent	AT: MICT, Treadmill interval RT: 10 Strength exercise	8 weeks	Individualised moderate	NR	3× per week for first 4 weeks. 5× per week for weeks 4–8	63.4%
Verheggen et al., 2021 [31]	0%	Aerobic	Cycle ergometer	8 weeks	65–85% HRR (increased over 55 min intervention)	55 min	2–4	98% compliance
Cronin et al., 2018 [32]	17%	Concurrent	AT: NR RT: 7 exercises	8 weeks	AT: RPE 5–7/10 RT: >70% 1RM	NR	3× per week	88%
Shukla et al., 2015 [33]	0%	Aerobic	Cycle ergometer	Single effort	Maximal intensity	ME = 11.72 ± 2.6 min C = 13.1 ± 3.4 min	NA	100%
Allen et al., 2018 [34]	22%	Aerobic	Cycle ergometer Treadmill	6 weeks	60–75% HRR	30–60 min	3 sessions	100% Compliance
Morita et al., 2019 [35]	9%	I = Aerobic C = ‘trunk muscle training’	I: brisk walk C: trunk exercise	12 weeks	I: >3 METs C: NR	60 min per session	I: daily C: 1× group session per week + daily home sessions	I: 97.1% attendance C: >90%
Rettedal et al., 2020 [36]	9%	Aerobic	Cycle ergometer	3 weeks	High-intensity	8–12 × 60 s bouts @ VO2 peak with 75 s recovery	9 sessions in total on non-consecutive days	100%
Taniguchi et al., 2018 [37]	6%	Aerobic	Cycle ergometer	5 weeks	60–75% VO2peak	30 min for weeks 1–2; 45 min for weeks 3–5	3 sessions	NR
Munukka et al., 2018 [38]	11%	Aerobic (interval)	Cycle ergometer	6 weeks	Low to moderate	40–60 min	3 sessions	NR
Cronin et al., 2019 [39]	12%	Concurrent	AT: NR RT: 7 exercises	8 weeks	AT: RPE 5–7/10 RT: >70% 1RM	NR	3 sessions	85%
Kern et al., 2020 [40]	32%	Aerobic	Bike: bike commute Mod: NR Vig: NR C: Habitual living	24 weeks	Bike: Not prescribed (commute) Mod: 50% VO2peak reserve Vig: 70% VO2peak reserve C: Not prescribed	Weekly energy expenditure of 1600 kcal for women and 2100 kcal for men	5 sessions	93%
Motiani et al., 2020 [41]	19%	Aerobic	Cycle ergometer	2 weeks	SIT: maximal effort interval MICT: 60% VO2peak	SIT: 4–6× 30 s bouts with 4 min recovery MICT: 40–60 min	3 sessions	NR
Warbeck et al., 2020 [42]	17%	Aerobic	Cycle ergometer Ellipticals Treadmills	12 weeks	HIIT (30 s of vigorous effort followed by 2 min of recovery)	60 min per session (HIIT for 15–35 min)	2 sessions	74.83% (attendance)
Dupuit et al., 2021 [43]	NR	Concurrent	Wattbike (HIIT) 10 resistance exercises (targeted whole body in circuit format)	12 weeks	AT: >85% HRmax (8 s high, 12 s recovery) RT: 8–12 rep max	AT: 20 min RT: ~25 min	3 sessions	97.5% attendance 99% compliance
Mahdieh et al., 2021 [44]	11%	Aerobic	Treadmill	10 weeks	Moderate (55–60% HRR in week 1) gradually increasing to high intensity by week 10 (70–75% HRR)	30 min in week one progressing to 45 min in week 10	3 sessions	89%
Mokhtarzade et al., 2021 [45]	17%	Concurrent	Aerobic: Jogging, running, cycling Resistance: Home based, 10 exercises	6 months	AT: periodised 50–65% HRR to 60–75% HRR RT: Periodised RPE 5–6 to 7–8	NR	5× per week (2× RT, 3× Aer)	90%

AT = Aerobic training, MICT = Moderate intensity continuous training, NR = Not recorded, NA = Not available, HIIT = High intensity interval training, LIT = low intensity exercise, HRR = Heart rate reserve, h = hours, Min = Minutes, ME = Myalgic encephalomyelitis, I = Intervention, C = Comparator, METs = Metabolic equivalents, VO_2_ = Volume of oxygen, AT = Aerobic training, RT = Resistance training, HRmax = Maximum Heart rate, RM = Repetition maximum, RPE = Rating of perceived exertion, Mod = Moderate intensity, Vig = Vigorous intensity and SIT = Spring interval training.

**Table 3:** Sample frequency and sequencing details.

Author	Method of Analysis	Number of Samples per Participant	Sample Collection Timepoints (Weeks)	Dietary Control (for Study Period)	Dietary Assessment	Taxonomic Labelling Tool Used
Craven et al., 2021 [18]	16S	4	0 (×2), 6 (AFT), 7 (Taper)	No	3-day diet diary at each testing point	NCBI database
Tabone et al., 2021 [19]	16S	2	0, <1	No	FFQ and 3 × 24-h food diary recall	Silva reference database
Zeppa et al., 2021 [20]	16S	2	0, 9	No	Daily diaries for duration of study (plus two weeks prior)	GreenGenes and UCLUST
Karl et al., 2017 [21]	16S	2	0, <1	Yes	No	RDP classifier
Zhao et al., 2018 [22]	16S	2	0, <1	Yes (type of food)	Questionnaire	Not Reported
Grosicki et al., 2019 [23]	16S	4	1, 19, 21 (after competition), 23	No	No	PAST: Paleontological Statistics Software
Keohane et al., 2019 [24]	metagenomic	4	0 (BEF), ~2, <5 (AFT), +3 months	No	FFQ (baseline), daily diet diary	MetaPhlAn2.0
Barton et al., 2020 [25]	metagenomic	14	Fortnightly (0–26)	No	daily diary—My Fitness Pal App	MetaPhlAn2 database
Oliveira et al., 2022 [26]	16S	2	0, <1		24-h food records	Kraken taxonomy + Bracken custom data base (GutHealth_DB)
Bycura et al., 2021 [27]	16S	28	−3, 0 (BEF), 8 (AFT), 11 (two samples per week)	No	No	Bayes classifier in q2-feature Classifier Genome Taxonomy Database
Erlandson et al., 2021 [28]	16S	2	0, 24	No	3-day diet diary	SINA
Resende et al., 2021 [29]	16S	2	0, 10	No	48 h food record, FFQ and 3-day food diary	Greengenes
Huber et al., 2019 [30]	NR	2 (*n* = 9)	0, 8	No	No	NR
Verheggen et al., 2021 [31]	16S	2	0, 8	No (recommended to not change dietary pattern)	24-h diary prior to sample, FFQ	NG-Tax
Cronin et al., 2018 [32]	metagenomic	2	0, 8	No	FFQ	Kraken taxonomy
Shukla et al., 2015 [33]	16S	3	0 (BEF), 48 h (AFT), 72 h		No	RDP classifier
Allen et al., 2018 [34]	16S	3	0 (BEF), 6 (AFT), 12 weeks	3-days prior to sample collection	3-day food menu was followed prior to each faecal collection. Menu organised from 7-day diet diary	RDP classifier
Morita et al., 2019 [35]	16S	2	0, 12	No	FFQ	Human Faecal Microbiota T RFLP profiling (10 groups)
Rettedal et al., 2020 [36]	16S	4	0 (x 2), 3	Recommended to not change dietary pattern	FFQ	SILVA database v.132
Taniguchi et al., 2018 [37]	16S	3	0, 5 (AFT), 10	No	Yes (Diet history questionnaire)	UCLUST
Munukka et al., 2018 [38]	Metagenomic and 16S	3	0, 6 (BEF), 12 (AFT)	No	3-day food diary	Silva 123.4 database
Cronin et al., 2019 [39]	Metagenomic	3	0, 8 (AFT), 16	No	No	Kaiju taxonomic assignment
Kern et al., 2020 [40]	16S	3	0 (BEF), 12, 24 (AFT)	No	3-day food diary	RDP classifier
Motiani et al., 2020 [41]	16S	2	0, 2	No	No	Greengenes GG 13.8 Database
Warbeck et al., 2020 [42]	16S	3	0, 12 (AFT, BEF for WLC), 24 (Follow up, AFT for WLC)	No	3-day diet diary	Silva 136 database
Dupuit et al., 2021 [43]	16S	2	0, 12	Recommended to not change dietary pattern	5-day food intake diary	Greengenes GG 13.8 Database
Mahdieh et al., 2021 [44]	16S	2	0, 10	No	72 h recall	Other: Targeted analysis of *Lactobacillus* and *Bifidobacterium*
Mokhtarzade et al., 2021 [45]	QPCR	2	0, 26	No	72 h recall	Other: Targeted analysis of *Prevotella*, *Akkermansia mucinophila*, *Faecalibacterium prausnitzii* and *Bacteroides*

NR = Not reported, BEF = Before intervention, AFT = After intervention, 16S = 16S mRNA (or DNA) sequencing, FFQ = Food frequency questionnaire, ME = Myalgic encephalomyelitis, Ex = Exercise, AT = Aerobic training.

**Table 4:** Key findings and recommendations for future research.

Findings and Recommendations	
*Exercise to modify the gut microbiota*	Higher-intensity and high-duration exercise appears important for microbiota change Adherence to exercise is necessary if changes seen with exercise, especially in untrained individuals, are to be maintained Aerobic and concurrent exercise interventions result in gut microbiota change; further research is required to determine the specific influence of resistance training
*Population influence*	Clinical and “apparently healthy” individuals both show similar changes in their gut microbiome in response to exercise training Available evidence suggests that taxonomic changes are different between groups, but functional outcomes may be similar
*Future research*	Attention to frequency, intensity, duration and type of exercise is required in future exercise–gut microbiota research Use of waitlist or crossover RCTs will provide a better evaluation of inter and intraindividual differences in the gut microbiota changes with exercise in humans Metagenomic analysis to evaluate functional changes is important for clarity in outcomes and to inform improved understanding of the exercise—gut microbiota relationship Research comparing and combining gut microbiota modifiers (such as diet and exercise) will optimise the clinical and practical interpretation of future data

## Data Availability

All data is available in supplementary tables.

## Supplementary Materials

The following supporting information can be downloaded at: https://www.mdpi.com/article/10.3390/nu15061534/s1, Table S1: Database Search Terms; Table S2: Delphi Quality Assessment; Table S3: Microbiota Outcomes.
